# Keyword index to Volume 85

**DOI:** 10.1054/bjoc.2001.2202

**Published:** 2001-12


British Journal of Cancer (2001) **85**(12), 2035–2039
© 2001 Cancer Research Campaign
doi: 10.1054/ bjoc.2001.2202, available online at http://www.idealibrary.com on IDEAL

### Keyword index to Volume 85

Accelerated chemotherapy 1444
Acquired resistance 1064
ADCC 463
Adenocarcinomas 1023, 1153, 1371, 1781
Adenomatous polyposis coli 69, 98
Adjuvant chemotherapy 827, 1251
Adjuvant therapy 1437
Advanced breast cancer 141
Advanced gastrointestinal cancer 1265
Aetiology 1667
Ageing 930
Aggressive fibromatosis 98
AIDS 1298
Alcohol 46, 341, 678
Alcohol intake 1700
ALIMTA 649
Allelic imbalance 1201
Alphafetoprotein 1119
Aminolevulinic acid 279, 727
Aminolevulinic acid derivatives 279
Aminolevulinic acid hexylester 727
5-Aminolevulinic acid 1572, 1794
5-Aminolevulinic acid esters 1572, 1794
Aminopeptidase N 924
Amsacrine 747
Androgen receptors 36, 1928
Androgen regulated transcripts 393
Angiogenesis 266, 439, 473, 863, 881, 1396, 2010
Angiogenesis inhibition 1540
Angiotensin 1396
Angiotensin II 1640
Anthracycline-containing regimen 141
Anthracycline/taxane resistance 798
Antiangiogenic therapy 584
Anti-apoptotic genes 405
Anti-cancer drugs 242
Antidotal activity 1964
Antimicrotubule agents 735
Antineoplastic drugs and medications 1400, 1611
Anti-p53 antibodies 1883
Antisense 1753
Anti-tumour activity 741
Apoptosis 115, 273, 279, 303, 453, 584, 764, 898, 902, 1047, 1403, 1522, 1564, 1764, 1771, 1781, 1801, 1914, 1964, 2004, 2017
AQ4N + cisplatin 625
Archival smears 398
Arimidex 317
Aromatase inhibitors 317
Asparagine synthetase 930
Assam 661
Asthma 1317
ATP 1987
Attenuated adenomatosis polyposis coli 523
Avascular necrosis 1624
Axillary surgery 668
5-Aza-2´-deoxycytidine 1351

Basal cells 422
Basal cell carcinomas 683, 1883
Basic fibroblast growth factor 1706
BAX 1359
Bax 1557
BCL-2 1359
Bcl-2 115, 273, 1557, 1564, 1764, 1781
Bcl-2 family 1801
bcl-2 phosphorylation 1403
Bcl-X 1557
Benign prostatic hyperplasia 576
Betel nut chewing 658, 661
BH3 115
Biochemotherapy 1871
Biomarkers 1952
Bioreductive drugs 1137
Birthweight 428
Bispecific antibodies 152
Bladder cancers 568, 1137, 1412, 1515, 1887
Bladder neoplasms 552, 977
Blood flow 1640, 1655
Bloom’s syndrome gene product 261
Body height 959
Body mass 1680
Body mass index 372
Body surface area estimation 23
Body weight 346
Body weight/body mass index 984
Bone resorption 78
Book reviews 1609, 1810, 1811
BQ123 1759
Brain tumours 285, 1396
BRCA1 538
BRCA1 36, 845, 850, 1201, 1675
BRCA1 gene 1347
BRCA2 36, 850, 1201, 1675
Breast cancer 1, 69, 78, 85, 171, 141, 225, 317, 372, 422, 428, 490, 538, 618, 637, 668, 798, 859, 869, 917, 959, 962, 1014, 1102, 1157, 1240, 1280, 1289, 1340, 1522, 1675, 1680, 1731, 1764 1878, 1914, 1958, 2017
Breast cancer cells 909
Breast cancer cell lines 902
Breast cancer prevention 1838
Breast cancer risks 36
Breast cancer therapy 147
Breast conservation therapy 668
Breast neoplasms 362, 1347
Breastfeeding 1685
Bromocriptine 1611

C225 584
c-KIT/SCF 405
Cachexia 297
Calman-Hine report 166
Camptothecin resistance 1077
Canada 1335
Cancer chemoprevention 618
Cancer chemotherapy 1219
Cancer gene therapy 1592
Cancer genetics services 166
Cancer genomics 1492
Cancer immunotherapy 1527
Cancer immunotherapy, human 1722
Cancer incidence 1332
Cancer patients 641
Cancer registry 367, 1251
Cancer screening 1332
Cancer vaccine 713
Cancer-testis antigens 713
Carbonyl reductase 1032
Carboxymethylbenzylamide dextran derivative 917
Carcinoembryonic antigens 1787
Carcinogenesis 1922
Carcinomas *in situ* 213
Carotenoids 977
Case-control studies 337, 341, 398, 683, 966, 991, 1326, 1667
Caspase 3 1522
Catechol oestrogens 859
Catechol-*O*-methyltransferase 859
β-Catenin 64, 98
γ-Catenin 64
Cathepsin S 1193
Caucasians 171
CCR1 891
CCRF-CEM 1400
CD4+ T cell clones 1527
CD44(var) 924
CDC25A 412
CDC25B 412
CDDP-based chemotherapy 1064
CDHP 939
*CDK4* genes 836
*CDKN2A* 527
*CDKN2A* genes 836
*CDKN2A* gene 1383
cDNA 1351
cDNA array 538
Cell cultures 285
Cell cycles 1771
Cell cycle arrest 898
Cell growth 1978
Cell lines 1014, 1492
Cell mediated immunity 473
Cell proliferation 1771
Cell scattering 1412
Cervical cancer 93, 398, 791, 966, 1153, 1675
CHART 1113
Chemokines 891
Chemopotentiation 625
Chemosensitivity 1425
Chemotherapy 649, 808, 816, 1099, 1240, 1444, 1456, 1646, 1850
Chemotherapy steroids 1624
Child development 1611
Childhood cancer 337, 350, 1680, 1685
Chk2 293
*CHK2* mutations 209
Chlorambucil 764
Chlorpromazine 1998
chLym-1 463
Chondrosarcomas 176
Chromosomal aberrations 1900
Chromosomal radiosensitivity 1157
Chromosome 10p15 1510
Chromosome 11 531, 1894
Chromosome 17 705, 1894
Chromosome aberrations 293
Chromosome instability 1887
Circulating tumour cells 431
Cisplatin 9, 823, 1124, 1206, 1456
Cleavable complex 1585
Cleavase fragment length polymorphism 845
Clinical features 538
Clinical resistance 509
Clinical trials 1432, 1467

Clonal evolution 182
Clonogenic assays 1585
CMF 1106
CNS toxicity 1233
Co-activators 1928
Co-localisation 909
Cohort studies 346, 362
Colchicine 1998
Collagenase 383
Colon cancer 772, 1251, 1437, 1572
Colon neoplasms 1047
Colonoscopy 972
Colorectal cancer 504, 523, 641, 692, 787, 827, 972, 1037, 1258, 1486, 1695, 1759, 1771, 1937
Colorectal liver metastases 1640, 1759
Colorectal neoplasms 346, 357
Colorectal tumours 764, 1492
Comparative genomic hybridization 213, 422, 697, 1492
Competing risks 1113
Computer touchscreens 1842
COMT 859
Consanguinity 1675
Consultations 1273
Continuous hyperfractionated accelerated radiotherapy 1113
Contralateral breast cancer 850
CpG-methylation 1168
Cremophor EL 1124, 1472
CTL epitope peptides 1722
$^{62}$Cu-PTSM 1640
Cyclin-dependent kinase inhibitors 1515
Cyclo-oxygenase-2 1023
Cyclophosphamide 798
Cytochrome P450 242
Cytoreduction 1824
Cytosols 85

Dacarbazine 1467
DC101 584
*DCC* 199
Death associated protein kinase family 1801
Decorin 228
Deletion mutations 176
Denaturing gradient gel electrophoresis 235, 523
Dermatomyositis 41
Dexamethasone 1099
Diagnosis 225, 656
Diarylsulfonylureas 1400
Diet 341
Dietary cholesterol 357
Differential displays 1162
Differentially expressed genes 393
Differentiated thyroid cancer 875
Differentiation 590, 1944, 2004
Diltiazem 1577
Dimethylbenz[*a*]anthracene 428
Dlk/ZIP kinase 1801
DNA damage 898
DNA methylatioin 1887
DNA microarrays 107
DNA ploidy 182
Docetaxel 242, 1247
Dorsal root ganglia 1219
Down-regulation 220
Doxorubicin 1964, 1987
Drug combinations 1077
Drug resistance 735, 902, 1175, 1425
Drug targeting 747
Ductal carcinoma 225, 422
dUTP 446
dUTPase 446
Dysplastic nevi 1304

Early detection 803
Early onset 859
EBAG9/RCAS1 1731
E-cadherin 1958
Efficacy 1326
EGFRvIII 1211
Eggs 357
Egypt 1037
ELISA 1193
ELISPOT 1738
Emetogenic chemotherapy 1099
EMT 1412
Endometrial cancer 333, 546
Endoscopy 972
Endothelial cells 584
Endothlin-1 1759
Enzyme-linked immunosorbent assay 1193
EO9 1137
EORTC QLQ-C30 1265
Epidemiology 962
Epidermal growth factor 303, 84
Epidermal growth factor receptors 1600
Epidermal growth factor receptor antagonists 1600
Epidermal growth factor receptor ligands 1412
Epirubicin 1106, 1456
Epithelial ovarian cancer 1032
Epitopes 875
Epstein-Barr virus 350, 997
ErbB2 705, 1787
$ET_A$ antagonists 1759
$ET_B$ antagonists 1759
Ethnicity 350
Etoposides 747
EUROCARE 787
Ewing's tumour 1646
Excess risk 362
Exercise 962
Exostoses 2 176
Expression 1162, 1211
Extracellular matrix 1968
Extracellular signal-regulated protein kinase-2 1403
Extraintestinal mucosa-associated lymphoid tissue lymphoma 1462
Extravasation 1387

Failure-specific prognostic factors 1113
Falkland Islands 1332
False negative cytology 398
Familial colorectal cancer 523
Familial melanoma 836
Familial papillary thyroid carcinoma 1831
Family history 674
Fas-L 1185
Fats 357
FcγR 463
Females 341
Fertility patterns 362
Fibroblasts 293
Fibroblast growth factor-8 576
Fine needle aspiration 1102
FISH 1201
Flexible modelling 795
Fluorescein-colchicine 1998
Fluorescence 1572
Fluorescence *in situ* hybridization 697, 1201
Fluoropyrimidine 439
5-Fluorouracil 798, 1258, 1937
Focal adhesion kinases 228
Folate 977
Follicular lymphomas 29
Follow-up studies 997
Founders 527
Fractionated administration 9
Fragile histidine triad genes 247
France 1664
Fusion transcripts 1535

α-GalCer 741
Gallbladder cancer 1922
GalNAc glycoproteins 1014
Gangliosides 285
$G_1$-arrest 293
Gastric adenoma 1147
Gastric cancer 199, 1147, 1317, 1322, 1585
Gastrointestinal cancer 1265
Gastro-oesophageal reflux 1317
Genes 1211
Gene amplification and expression 518
Gene clones 1162
Gene expression 107, 266, 1206, 1372
Gene mutations 64
Gene overexpression in cancer 190
Gene therapy 1432
Genetics 1499
Genetic analysis 687
Genetic counselling 166
Genetic polymorphism 859
Genistein 618
Germ cell tumours 823, 1119
Germ line mutations 523, 836
Glioblastoma 518
Gliomas 55, 1185, 1396
Glomerular filtration rates 325
Glucocorticoids 683, 1611
GM-CSF 152, 1444
Good practices 1251
gp 100/pmel17 1738
$G_2$-phase 1157
G-protein expression 758
Granisetron 1099
Granulocyte-macrophage colony-stimulating factor 1467
Growth hormone antagonists 428
Growth inhibition 453
Growth kinetics 490
Gynaecological oncologists 1824

Haemangioma 362
Haematolymphoproliferative disorders 997
*Haemophilus influenzae* type b 337
Head and neck cancer 630, 1055
Head and neck squamous cell carcinoma 649
Health service organisation 166
Health services accessibility 497
Height 1680
*Helicobacter pylori* 1322
*Helix pomatia* lectin 1014
Helper T cells 1738
Heparan sulfate 1094
Hepatocarcinogenesis 697
Hepatocellular carcinoma 228, 697, 1162, 1850
HER2 152
HER-2/neu 1764
HER-2/neu peptide 1527
Hereditary breast cancer 209, 845
Hereditary multiple exostoses 176
Hereditary non-polyposis colorectal cancer 1486
Herpesvirus 8, human 379
Heterogeneity 182
High throughput screening 1206
High-dose chemotherapy 484, 1240
High-dose oestrogens 147
Histology 1347
HIV 1298
hMLH1 1147
hMSH2 1147
Hodgkin's disease 350
Homing 1387
Hormone replacement therapy 674
HPA 1014
HPMA copolymer 1070
HPV-infection 1153
hTERT 752
hTR 1510
hTRF1 752
Humans 171, 1372, 1700
Human herpesvirus 8 1298
Human papillomavirus 966, 1551
Humoral immunity 473

 *© Cancer Research Campaign 2001*

Hyaluronan receptors 600
Hyaluronan synthase 600
Hypermethylated in cancer gene-1 1878
Hypermethylation 69, 930, 1878
Hypersensitivity pneumonitis 1247
Hypomethylation 563
Hypoxia 891, 1577
Hypoxia inducible factors 881

I1307K APC mutation 1368
IgG uptake 1968
IgGκ Fab 875
Immune evasion 1047
Immunocytochemistry staining 1713
Immunohistochemistry 576, 1193, 1731, 1922, 1937
Immunoluminescence 924
Immunophototherapy 1787
Immunosuppression 997
Immunosuppressive therapy 683
Immunotherapy 152, 713
*In situ* hybridization 383, 869, 1510
Incidence 1322, 1335
Information and support requests 641
Inherited predisposition to cancer 1368
Initial therapies 29
Insulin 346
Insulin-like growth factor binding protein-3 74, 1695
Insulin-like growth factor system 147, 1838
Insulin-like growth factor-1 74, 428, 991, 1695
Insulin-like growth factor-1R inhibitors 2017
Insulin-like growth factor-2 1695
Intercellular adhesion molecule-1 924, 1351
Interferon 29
Interferon-α 1130
Interferon-α sensitivity 107
Interferon-γ 741
Interleukin-1 receptor antagonists 1600
Interleukin-2 1130
Interleukin-6 518
Inter-observer variability 1634
Interstitial fluid pressure 1968
Intraperitoneal topotecan 1627
Intrauterine exposures 959
Intravital microscopy 431
Invasion 122, 869, 1600
Ionizing radiation 293, 362
Irinotecan 9
Irradiation 1055
Isolated limb infusions 157
Isotretinoin 630
Isotypes 735
Italy 379

JNK 303

Kallikrein 220
Kaposi's sarcoma 379, 1298
Kaposi's sarcoma herpesvirus 1298
Keratin 14 422
Keratinocytes 630
Ki-67 1106, 1781
Kidney neoplasms 984
Ki-ras 692
Kirsten ras mutations 692
KLK10 220
Knockout mouse 431

Laminin-1 1387
Laminin-5 383
Langerhans cells 1944
Laryngeal neoplasms 678
Laser capture microdissection 235
Letters to the Editors 137, 139, 140, 780, 781, 1418, 1606, 1607
Leucaphereses 1713
Leucovorin 798
Leukaemia 337
Leuprolide 333
Li-Fraumeni syndrome 293
Li-Fraumeni-like syndrome 209
LINE-1 1887
Lipid-mobilizing factor 758
Lipolysis 758
Liposomal doxorubicin 1850
Liver 1540
Liver metastases 504, 612
Liver microcirculation 431
Lombardia 379
Losartan 1396
Loss of heterozygosity 531, 1201, 1351, 1510, 1894
Loss of heterozygosity, 1*p36* 204
Low-incidence areas 721
67LR 1387
Lugol 1006
Lung cancer 235, 255, 1326
Lung neoplasms 678
Lung tumours 1193
Luteinizing-hormone releasing hormone analogues 333
LY231514 649
Lycobetaine 1585
Lymph node metastases 93, 255, 1032
Lymphatic mapping 791
Lymphocytes 1731
Lymphocytic thyroiditis 1831
Lymphoma 463, 930, 1462
Lymphopenia 816
Lymphoscintigraphy 791

Macrophages 285
*MAGE* 713
MAGE-A3 1340
Magnetic resonance imaging 1655
Maintenance 29
Major histocompatibility complex-II 1193
Malignancy 1611
Malignant cell growth 618
Malignant melanomas 1871
Malignant mesothelioma 600, 863
Mammary carcinoma 546
Mammary glands 1522
Mammographies 225
Management 808
Mantova 379
Mapping 1510
Marimastat 1865
Mastectomy 668, 1952
Material deprivation 350
Matrigel 590
Matrix metalloproteinases 1865
Matrix metalloproteinase-1 1600
Matrix metalloproteinase-2 1706
Matrix metalloproteinase-9 1706
Maturation 1944
*MBD2* 1168
MCF-7ras 917
MDA-MB-231 1978
MDM2 721, 1746
MDR 1987
MDR-1 1175
*mdr-1* 1746
Meat 357
*MeCP2* 1168
Mediterranean populations 1304
Medroxyprogesterone acetate 1
Medullary thyroid carcinoma 1546
Medulloblastoma 705, 1801
Melanomas 107, 157, 527, 741, 803, 1304, 1467, 1738
Melanoma, familial 836
Melanoma, human 1944
Melanoma thickness 1304
Melphalan 157
Men 641
Menopause 346
Meta-analysis 984, 991, 1700
Metabolism 630
Metalloproteinases 55, 122
Metastases 78, 285, 608, 1014, 1937
Metastatic germ cell tumours 823
Metastatic lesions 313
Metastatic non-small-cell lung cancer 1452
Methodology 1655
Methylation 199
Methylation-specific polymerase chain reaction 69
MHC alleles 1527
Microdialysis 157
Micrometastases 490, 1340
Microsatellite instability 568, 1037, 1064, 1147, 1486
Microsimulation models 1280
Migration 1387
Minisatellite 1486
Mismatch repairs 568
Mitochondria 1801
Mitogen-activated protein kinase 303, 1175, 1403
Mitomycin C 1137
*MN/CA9* 563
Molecular and cellular mechanisms 1233
Molecular diagnostics 1546
Molecular profiles 538
Molecular-based diagnosis 102
Monoclonal antibodies 875, 1193, 1787, 1964
Monofunctional ZD2767 analogues 764
Monozygotic twins 493
Morbidity 325
Morphology 1535, 1978
Mortality 803, 1664
Mouse 930
MRP1 1564
MSH3 568
MSH6 568
Mucositis 1964
Multicell tumour spheroids 727
Multidrug resistance 1998
Multiple myeloma 1387
Multitargeted antifolate 649
Multivariate analysis 509
Muscle proteolysis 297
Mutational analysis 845
Mutations 199, 1383
MVR-PCR 1486
*MYC* 705
MYCN 531
Myeloma 325
Myoepithelial 422

NADH oxidase 1400
Neoplasms 261, 1700
Neuraminic acid 285
Neuritic differentiation 1564
Neuroblastomas 182, 493, 531, 1564, 2004
Neurotoxicity 1219
Neutrophils 463
Newborns 1295
Nipple aspirate fluid 1952
Node-negative primary breast cancer 795
Non-astrocytic tumours 204
Non-Hodgkin's lymphoma 1298, 1425, 1900
Non-melanoma skin cancer 683
Non-platinum-based regimen 1452
Non-seminomatous germ cell tumours 608
Non-small-cell lung cancer 9, 14, 129, 881, 939, 1113, 1247, 1456, 1706
Normal cytology 398

Normal epithelial cell-specific 1 genes 220
Norway 959
Nottingham prognostic index 795
NQ01 1137
Nucleolus 1219
Nude rat 2004
Nurse-led clinics 1853
Nutrition 959

Obesity 984
Odds ratio 991
Oesophageal cancer 341, 658, 661, 721, 1006, 1317, 1499, 1667, 1671, 1781
Oestradiol 317
Oestrogen 317, 1731
Oestrogen receptors 372, 546
Older women 1289
Oligodeoxynucleotides 1753
Oncogenes 697
Oncologists 1634
Oncology 1842
Ondansetron 1099
Oral administration 1472
Oral cancer 46
Oral cavity neoplasms 678
Oral squamous cell carcinoma 122
Oral topotecan 1124
Orthotopic xenografts 608
Osteoclasts 78
Osteosarcoma 1968
Outcome 325
Ovarian adenocarcinoma 1351
Ovarian cancer 190, 313, 687, 891, 944, 1064, 1124, 1175, 1359, 1540, 1592, 1627, 1753, 1824
Ovarian tumours 247
Overall survival 944
Oxaliplatin 1258

*P15* 1515
*p16* gene 1383
*p21* 1359
1*p36* 204
p42/p44 kinases 1978
p53 293, 1102, 1153, 1499, 1764, 1781
*p*53 898, 902, 1878
p53 protein 1937
p53 tumour suppressor 1813
*P6*3 721
p73 1499, 1551
*p73* gene 204
Paclitaxel 141, 303, 902, 1472
Paediatric oncology 23
Pain 497
Pain measurements 497
Palliation 1265
Palliative care 816, 1478
Pancreatic cancer 612, 1865
Pancreatic cancer cells 752
Papillomavirus, human 398
Parathyroid-hormone-related protein 1722
Parotid glands 1055
Past use 674
Patients 1634
Patient participation 1273
Patient prognosis 313
Patterns of care 1251
Pemetrexed disodium 649
Pellet extracts 85
Pentoxifylline 1577
Pepsinogen C 228
Peptides 115
Performance status 1634
Peripheral benzodiazepine receptors 1771
Peripheral blood stem cells 1119
Peru 966
P-glycoprotein 1746, 1998
Pharmacogenetics 1425
Pharmacogenomics 107
Pharmacokinetics 1627
Phase I studies 1627
Phase II studies 9
Phenotypes 1368
Photodynamic therapies 279, 1794
Phthalocyanine 1787
Physical activity 962, 1311
PI-3K 303
Pigmentation 1304
Plasma membrane 1400
Plasminogen binding 909
Platelet-derived growth factor 1706
Platinum drugs 1219
Ploidy 14
Pluronic block copolymers 1987
Po Valley 379
Poisson regression 1335
Poliovirus vaccines 1295
Poly(ADP)ribose polymerase 1403
Polymer prodrugs 1070
Polymer-directed enzyme prodrug therapy 1070
Polymer-enzyme conjugates 1070
Polymerase chain reaction 1546
Polymorphisms 36, 171, 827, 1153, 1499, 1504
Polymyositis 41
Population health impacts 1280
Population studies 1347
Positron emission tomography 1640
Preclinical pharmacology 484
Prediction of response 1871
Predictive factors 1478
Pre-mRNA splicing 190
Present use 674
Prevention 428, 1280
Primary brain lymphomas 204
Primary breast cancer 74, 85, 1958
Profiles 1211
Progesterone receptors 372, 546
Prognosis 14, 85, 313, 367, 412, 552, 705, 808, 831, 863, 881, 1032 1359, 1535, 1557, 1878
Prognostic factors 1646, 1671
Prognostic indicators 1958
Prognostic models 944
Prognostic values 1634
Progression 869
Proliferation 772
Proliferation status 727
Prophylactic treatment 1055
Prostaglandins 1023
Prostate androgen regulated transcript 1 393
Prostate cancer 115, 152, 393, 453, 557, 576, 641 656, 991, 1504, 1722, 1928
Prostate spheroids 590
Prostate-specific antigens 656
Prostate-specific antigen mRNA bearing cells 557
Prostatic neoplasms 497
Protease-activated receptor-2 772
Proteins 357
Proteoglycans 1094
Proteolysis 74
Proteolysis-inducing factor 297
Protoporphyrin IX 727, 1572
Protracted venous infusion 1258
PR-transfection 1978
P-selectin 431
Psychological distress 1842

Quality of life 497, 1265, 1478
Quantitative reverse transcriptase polymerase chain reaction 393

RAC 3 1928
Radiation 1611, 1853
Radiosensitivity 1577, 2010, 2017
Radiotherapy 1233, 1671
Raf 1753
Ras oncogenes 235
RASCAL II Study 692
RCAS1 1922
Real-time quantitative reverse transcription-polymerase chain reaction 102
Receptors 171, 584, 1023
Receptor subtypes 266
Recombinant adeno-associated virus 1592
Recombinant adenovirus 1592
Record linkages 41
Rectal cancer 1437
Regional chemotherapy 157
Regional intra-arterial treatment 504
Regional variations in survival 637
Registries 497
relA 1914
Renal cancer 1130
Renal cell carcinoma 563, 924, 953, 1372
Resistance 1746
Ret oncogene activation 1831
Retinoblastoma protein family 546
Retinoic acid 122, 2004
Retinoic acid receptors 453
Retinoic acid receptor antagonists 453
Retinoids 630
Reverse transcription-polymerase chainreaction 55, 405, 557, 1119, 1546, 1713
Rhabdomyosarcoma 831, 1746
Ribozymes 1185
Risk factors 428, 816, 972, 984, 1304, 1499, 1700
Rituximab 1619
RNA polymerase 190
Routine practices 1842
RT-PCR 1340
RU486 morphology 1978
S-1 939
Salvage chemotherapy 509, 798
Schistosomiasis 1037
SCID 608
Scintigraphy 1462
Screening 656, 1289, 1326, 1842
Second malignancies 997
Second-line chemotherapy 1456
Selective oestrogen receptor modulators 1838
Sensitisation 1987
Sentinel lymph nodes 791, 1340
Sequential chemotherapy 141
Serine protease 772
Serine/arginine-rich proteins 190
Serology 337
Serous carcinoma 247
Sexual habits 46
Sialic acid 285
Side-effects 1853
Sigmoidoscopy 972
Signal transduction 618
Skin tumours 1883
Small-cell lung cancer 808, 1444, 1713
Smoking 341
Sodium phenylacetate 917
Solid tumours 484
Soluble Fas ligands 1047
Somatostatin 266, 1462
Somatostatin receptors 266
Soya 618
Soyfood 372
Splicing factors 190
Sporadic renal cell carcinoma 1226
Squamous-cell cancer of the head and neck 1383

*© Cancer Research Campaign 2001*

Squamous-cell carcinoma 630, 658, 683, 721, 1006, 1883
Squamous cell carcinoma of the oesophagus 412
Squamous papillomas 1794
SR family 190
SR-A1 190
Stages 787
Staging 863
Superantigens 129
Supportive care 1853
Suppression subtractive hybridization 228, 1206
Surgery 1824
Surgery signals 490
Survival 367, 576, 637, 787, 808, 1251, 1492, 1850, 1900
Survival outcomes 1928
Survival rates 1671
Susceptibility 1504
SV40 1295
Synergism 917
Synovial sarcoma 405, 1535

Tamoxifen 317, 1106, 1130, 1280
Taxol 1175
tcf/lef transcription factors 98
Tegafur 939
Telomerase 752, 1006
Telomerase repressor genes 1510
Telomere 898
Telomeric restriction fragment 752
Tenascin-C 383
Testicular cancer 213, 220, 1624
Testicular germ cell tumours 608
Thalidomide 953
Thymidine phosphorylase surexpression 439
Thymidylate synthase 446, 827, 1937
Thyroglobulin 102
Thyroid cancer 102, 875, 1335
Thyroid carcinoma, familial papillary 1831
Thyroid peroxidase 875
Time trends 1335
Tissue inhibitors of metalloproteinases 55
Tissue-type plasminogen activators 122
Tobacco 46
Tobacco chewing 661
Topoisomerase IIa 747
Topoisomerase IIb 747, 1585
Topoisomerase inhibitors 1077
Topotecan 1627
Toxicity 1850, 1865
*TP53* 721, 1359
Transfection 735
Transforming growth factor-β 687
Transforming growth factor-$\beta_1$ 612
Transitional cell carcinoma, human 1515
Transitional cell carcinomas of the urinary bladder 1894
Translocation 831
Treatment-related deaths 816
Trends 1664
TRF1 898
TRF2 898
Trypsin 772
*TSC1* 1226
*TSC2* 1226
Tubulin 735
Tumorigenesis 1185
Tumorigenicity 1914
Tumours 1655
Tumour angiogenesis 439
Tumour cell contamination 1713
Tumour dormancy 490
Tumour growth control 2010
Tumour incidence 674
Tumour markers 393, 552
Tumour perfusion 1577
Tumour spreading 600
Tumour suppressor genes 220, 697, 1887
Tumour targeting 1070
Tumour therapy 1540
Tumour-infiltrating lymphocytes 1722, 1871
Tumour-rejection antigens 713
Tumoural markers 1883
Tyrosinase 1738

Ubiquitin-proteasome proteolysis 297
Ultraviolet radiation 1504
Ungeremine 1585
uPARs 85, 924
Uracil misincorporation 446
Urokinase 909
Urokinase-type plasminogen activators 85, 924
Urologic neoplasms 977
Uterus 1023

V126D 527
Vaccines 1527, 1738
Vascular endothelial growth factor 273, 313, 576, 584, 612, 795, 1706
Vascular endothelial growth factor receptors 255
Vascular endothelial growth factor-C 93, 255
Vascular endothelial growth factor-dependent proliferation 2010
Vascular endothelium 266
Vasculature 1968
Vinblastine 1998
Vinorelbine 1403
Vitamin-A 977, 2004
Vitamin-C 977
Vitamin-D 171
Vitamin-E 977
VM mouse 285
Vα24+NKT-cells 741
Vulval cancer 1551

Western blotting 1102
Wilms’ tumour 1557

X-irradiation 752

ZD2767 764
ZD9331 446

*© Cancer Research Campaign 2001*

